# Emergent stochastic oscillations and signal detection in tree networks of excitable elements

**DOI:** 10.1038/s41598-017-04193-8

**Published:** 2017-06-21

**Authors:** Justus Kromer, Ali Khaledi-Nasab, Lutz Schimansky-Geier, Alexander B. Neiman

**Affiliations:** 1 Center for Advancing Electronics Dresden, TU Dresden, Mommsenstrasse 15, 01069 Dresden, Germany; 2 Department of Physics and Astronomy, Ohio University, Athens, Ohio, 45701 USA; 3 Department of Physics, Humboldt-Universität zu Berlin, Newtonstrasse 15, 12489 Berlin, Germany; 4 Bernstein Center for Computational Neuroscience, Berlin, Germany; 5 Neuroscience Program, Ohio University, Athens, Ohio, 45701 USA

## Abstract

We study the stochastic dynamics of strongly-coupled excitable elements on a tree network. The peripheral nodes receive independent random inputs which may induce large spiking events propagating through the branches of the tree and leading to global coherent oscillations in the network. This scenario may be relevant to action potential generation in certain sensory neurons, which possess myelinated distal dendritic tree-like arbors with excitable nodes of Ranvier at peripheral and branching nodes and exhibit noisy periodic sequences of action potentials. We focus on the spiking statistics of the central node, which fires in response to a noisy input at peripheral nodes. We show that, in the strong coupling regime, relevant to myelinated dendritic trees, the spike train statistics can be predicted from an isolated excitable element with rescaled parameters according to the network topology. Furthermore, we show that by varying the network topology the spike train statistics of the central node can be tuned to have a certain firing rate and variability, or to allow for an optimal discrimination of inputs applied at the peripheral nodes.

## Introduction

Coupled noisy excitable systems serve as relevant models for a wide range of natural phenomena, including pattern formation in chemical reactions^1, 2^ and in social networks^3–6^, dynamics of gene regulatory networks^7^ and of single and networked neurons^8–10^. In particular, synchronization of neuronal activity on the level of neural networks has been studied extensively^11–13^ using models of coupled excitable systems^14–16^. Networks of noisy excitable elements exhibit a rich variety of spatio-temporal dynamics, depending on the strength and topology of coupling and the noise intensity^17–21^. For example, the coherence of emergent network oscillations can be controlled by modifying the noise intensity, the coupling strength, or by changing the network size or topology^22–27^. The dynamic range and sensitivity of complex networks of excitable elements to external stimuli can by optimized for critical topologies^28–30^.

**Figure 1:**
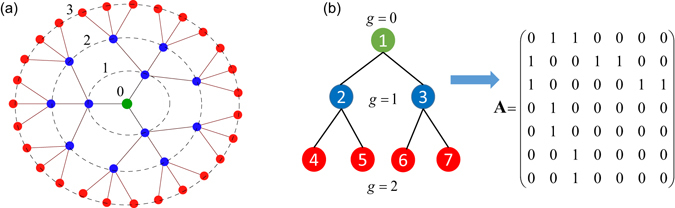
(**a**): Tree network with branching order, *d* = 3, and *G* = 3 generations. Peripheral nodes are marked red and receive external excitations. Dashed circles indicate corresponding shells of the tree’s generations, *g* = 1, 2 and 3; generation *g* = 0 refers to the central (primary branching) node (green). For a discrete cable model of myelinated dendrites, active elements are nodes of Ranvier, which are connected by passive resistors. (**b**): Adjacency matrix **A** (right) of a tree network with branching order *d* = 2 and two generations, *G* = 2 (left). Numbering of nodes starts with the central node and ends with the peripheral nodes.

In the present paper, we focus on the dynamics of regular tree networks of strongly coupled excitable elements which receive random and independent excitations to their peripheral nodes, as sketched in Fig. 1(a). Our study is motivated by the morphology of certain peripheral sensory neurons, which possess branched myelinated segments at their distal endings, with multiple nodes of Ranvier. Their extended terminal branching resembles the dendrite structure of neurons in the central nervous system (CNS)^31, 32^. Myelinated segments form a tree-like structure with nodes of Ranvier at each branching point.

Myelination terminates at peripheral nodes of Ranvier, called heminodes, which receive sensory signals. Thus, such sensory neurons may possess multiple spike initiation zones at heminodes which encode a local sensory signal into a stream of action potentials (APs) which are merged into a single output spike train transmitted to the CNS^33^. Examples for such neurons are the afferent innervation of muscle spindles^34–37^, pain receptors^38^, cutaneous mechanoreceptors^39, 40^, and lung receptors^41^. Interestingly, sensory neurons with myelinated dendrites may exhibit spontaneous activity characterized by coherent periodic spiking, despite that their peripheral heminodes presumably receive uncorrelated noisy excitations^36^. Figure 1(a) then can be viewed as a model for a branched myelinated dendritic terminal, where peripheral nodes receive uncorrelated stochastic inputs and are linked by myelinated segments. Due to the high density of Na^+^ ion channels at the nodes of Ranvier, APs may be excited independently at different peripheral nodes. High electrical conductivity of myelinated segments, which link the individual nodes, result in a strong coupling between the nodes. Therefore, their stochastic dynamics synchronizes. This may result in noisy periodic spiking of the primary branching (central) node, as we have shown for star networks of excitable elements^42^.

Here we use a biophysical model for nodes of Ranvier connected by myelinated links on regular trees and show numerically and analytically that the collective response of the network can be deduced from the stochastic dynamics of a single effective node with parameters scaled according to the network size and topology. Thus, our study allows for the prediction of the stochastic network dynamics from the tree topology. We then discuss how the tree topology affects the firing statistics of the central node and the discriminability of input signals.

## Model and Methods

### Discrete Cable Model

In the present paper, we study the stochastic dynamics of excitable elements linked on a regular tree (see Fig. 1). Branching starts at the primary (central) node and continues through several generations. Only the peripheral nodes receive external inputs. Referring to a model of branched myelinated dendrites, these peripheral nodes are called heminodes and receive inputs from thin unmyelinated processes (neurites). APs are initiated at the heminodes and then propagate on the tree towards the primary branching node and eventually to the CNS.

Here we consider regular trees whose topology is characterized by two parameters: the branching, *d*, and the number of generations, *G*. Given these two parameters, the total number of nodes, *N*, and the number of peripheral nodes, *N*_*p*_, are given by

1 $$N=\frac{{d}^{G+1}-1}{d-1}\,{\rm{and}}\,{N}_{p}={d}^{G},$$

respectively. The dynamics of the membrane potential is approximated by a discrete cable model^43^ in which nodes of Ranvier are connected by passive resistive links according to the network topology. All active nodes and passive links are assumed to be identical, except that peripheral nodes receive external inputs. The membrane potential *V*_*k*_(*t*) of the *k* th node obeys the dynamics

2 $$C{\dot{V}}_{k}=-{I}_{{\rm{i}}{\rm{o}}{\rm{n}}}[{V}_{k},{{\bf{u}}}_{k}]+\kappa \sum _{j=0}^{N-1}{A}_{k,j}({V}_{j}-{V}_{k})+{I}_{{{\rm{e}}{\rm{x}}{\rm{t}}}_{{\rm{k}}}},$$

$${\dot{{\bf{u}}}}_{k}={\bf{a}}({V}_{k}\mathrm{)(1}-{{\bf{u}}}_{k})-{\boldsymbol{\beta }}({V}_{k}\mathrm{)(1}-{{\bf{u}}}_{k}),$$

where the index *k* = 0, 1, 2, …, *N* − 1 marks the respective node. In particular, *k* = 0 refers to the central node. In Eq. (2) the term ***I***_ion_[*V*_*k*_, **u**_*k*_] stands for nodal ionic currents and **u**_*k*_(*t*) is a vector whose components are the gating variables of the nodal ion channels and *C* = 2 *μ*F/cm^2^ is the nodal capacitance per area. In the following we use two particular models for the nodes of Ranvier: a Hodgkin-Huxley-type (HH) model with Na^+^ and leak currents^42^ and the Frankenhaeuser-Huxley (FH) model which includes additional K^+^ and persistent Na^+^ currents. The HH nodal model includes two gating variables, *m* and *h*, for Na^+^ channels, i.e. ***I***_ion_[*V*, **u**] = ***I***_ion_[*V*,*m*,*h*]. The FH model includes two additional gating variables, *n* for K^+^, and *p* for persistent Na^+^ channels: ***I***_ion_[*V*, **u**] = ***I***_ion_[*V*,*m*,*h*,*n*,*p*]. The detailed equations and parameters of the nodal models are provided in the Supplementary Material.

The coupling term in Eq. (2), $\kappa {\sum }_{j=0}^{N-1}{A}_{k,j}({V}_{j}-{V}_{k})$, contains the adjacency matrix **A** of the undirected tree graph, which is a *N* × *N* symmetric matrix with elements *A*_*i*,*j*_ = 1 for connected nodes *i* and *j*, and *A*_*i*,*j*_ = 0 for unconnected nodes. Figure 1(b) shows an example of the adjacency matrix. The coupling strength, ***κ***, can be calculated from the sizes of the node and myelinated links, and the axoplasmic resistivity:

3 $$\kappa =\frac{a}{4lL\rho },$$

where *a* is the diameter of the node (and of links), *l* is the nodal length, *L* is the length of connecting links and *ρ* is the axoplasmic resistivity. For example, for *ρ* = 100 Ωcm, the nodal diameter and length *a* = 10 *μ*m, *l* = 1 *μ*m, and the length of myelinated segment *L* = 200 *μ*m, the coupling strength is *κ* = 1250 mS/cm^2^. This provides a biophysically-plausible range for *κ*, which we use as a control parameter in the following.

The external current *I*_ext_ is applied only to the peripheral nodes and consists of a constant part *I* and noisy part, i.e.

4 $${I}_{{{\rm{e}}{\rm{x}}{\rm{t}}}_{{\rm{k}}}}={\delta }_{k,p}[I+\sqrt{2D}\,{\xi }_{p}(t)],$$

where *p* denotes indicies of peripheral nodes; *δ*_*k*,*p*_ is the Kronecker delta; *D* scales the intensity of the Gaussian white noise *ξ*_*p*_(*t*), which is uncorrelated for different peripheral nodes, 〈*ξ*_*i*_(*t*)*ξ*_*j*_(*t* + *τ*)〉 = *δ*_*i*,*j*_ *δ*(*τ*). Thus, peripheral nodes receive random uncorrelated inputs.

Equations (2) were integrated numerically using explicit Euler-Maruyama methods with timestep of 0.1 *μ*s.

### Synchronization and Variability of Generated Sequences of Action Potentials

A spike is identified as a full-size AP with a magnitude of at least 60 mV. We extracted a sequence of spike times, ${t}_{j}^{(k)}$, at the *k*-th node in the network from 60–120 s long simulation runs. In order to study synchronization of the nodes, we calculated their instantaneous phases as,

5 $${\varphi }_{k}(t)=2\pi \frac{t-{t}_{j}^{(k)}}{{t}_{j+1}^{(k)}-{t}_{j}^{(k)}}+2\pi j,\quad {t}_{j}^{(k)} < t < {t}_{j+1}^{(k)},$$

where the index *k* refers to the *k*-th node. Thus, *φ*_*k*_(*t*) increases by 2*π* at every spike of the *k*-th node and interpolates linearly between consecutive spikes. The degree of synchronization is measured by the time averaged Kuramoto order parameter:^44^

6 $${\rho }_{n}=\overline{|{\langle {e}^{I{\varphi }_{i}}\rangle }_{n}|},$$

where the bar stands for long-time averaging and the angular brackets, 〈⋅〉_*n*_, denote averaging over a set of nodes *n*. The Kuramoto order parameter *ρ*_*n*_ quantifies the degree of synchronization of a set of nodes *n*: *ρ*_*n*_ = 1 refers to perfectly-synchronized nodes, while *ρ*_*n*_ = 0 to complete asynchrony. In the following we study synchronization of two sets of nodes. Synchronization of the peripheral nodes is quantified by *ρ*_P_ , i.e. averages are taken over peripheral nodes only, while the synchrony of the entire network is quantified by *ρ*_C_, i.e. averages are taken over all nodes.

Our primary interest is the statistics of a spike train generated by the central node. The corresponding sequence of interspike intervals (ISIs) ${\rm{\Delta }}{t}_{j}={t}_{j+1}^{(1)}-{t}_{j}^{(1)}$, is characterized by the mean firing rate, *r* and the coefficient of variation (CV), *C*_*V*_ as,

7 $$r=\frac{1}{\langle {\rm{\Delta }}{t}_{j}\rangle },\quad {C}_{V}=r\sqrt{\langle {({\rm{\Delta }}{t}_{j}-\langle {\rm{\Delta }}{t}_{j}\rangle )}^{2}\rangle },$$

where the average is taken over all ISIs in the spike train of the central node.

### Signal Detection

To characterize the signal detection capacity of a tree network, we considered a small constant stimulus, Δ*I*, applied to the peripheral nodes in addition to the stimulus *I*, and calculated a normalized distance between resulting spike count distributions of the central node with and without this addition. Such a measure of distance is given by the discriminability, *d*′, which in the case of a Gaussian spike count distribution is defined as^45^,

8 $$d^{\prime} =2\frac{|{\mu }_{T}(I)-{\mu }_{T}(I+{\rm{\Delta }}I)|}{{\sigma }_{T}(I)+{\sigma }_{T}(I+{\rm{\Delta }}I)},$$

where *μ*_*T*_ and *σ*_*T*_ are the mean and standard deviation of the spike count in a time interval *T*, respectively. The discriminability quantifies how well the network responses to two different stimuli, *I* and *I* + Δ*I*, can be distinguished by observing corresponding spike count statistics at the central node. Note that *d*′ defined by Eq. (8) quantifies the discriminability of two signals according to observed spike count distributions only, and does not account for stimulus discrimination on the base of other types of statistics, e.g. variability of interspike intervals.

The discriminability is related to the Fisher information, which provides the theoretical limit of how accurately a stimulus *I* can be estimated by observing a spike train^46^. For the spike count statistics, a lower bound of the Fisher information can be written as^47^,

9 $${J}_{{\rm{LB}}}(I)=\frac{1}{{\sigma }_{T}^{2}(I)}\,{(\frac{d{\mu }_{T}}{dI})}^{2},$$

and is related to the discriminability, *d*′ by^47^,

10 $$d^{\prime} \approx {\rm{\Delta }}I\sqrt{{J}_{{\rm{LB}}}(I)}.$$

Larger values of the Fisher information refer to more accurate estimation of the stimulus from the spike train and a better discrimination between two stimuli *I* and *I* + Δ*I*. In the following we will show that in the strong coupling limit, a tree network can be reduced to a single node with an effective input stimulus current and noise. The lower bound of the Fisher information will be computed for the single node and then linked and compared to discriminability of the tree network using Eq. (10).

The discriminability Eq. (8) was calculated by collecting spike counts of the central node for 5000 independent time intervals of lengths *T* = 200 ms, and calculating the mean and standard deviation for two values of the stimulus, *I* and *I* + Δ*I*, applied to the peripheral nodes of a tree network^47^. We also calculated the lower bound of the Fisher information Eq. (9) for the single uncoupled node as a function of the input current (stimulus) *I* and the noise intensity, *D*, using a similar numerical procedure.

## Results

### Emergence of Periodic Firing in Deterministic Tree Networks

At first, we consider the case of a deterministic input, *D* = 0. In the absence of external input, *I*_e*xt*_ = 0, an isolated node is in the excitable regime. A sufficiently high constant current, *I*_AH_ results in a subcritical Andronov-Hopf bifurcation of the equilibrium state rendering an isolated node to fire a periodic sequence of APs. The corresponding limit cycle disappears in a saddle-node bifurcation for a lower external current, *I*_SN_. For the HH nodal model the saddle-node bifurcation occurs at *I*_SN_ ≈ 28.15 *μ*A/cm^2^ and the subcritical Andronov-Hopf bifurcation at *I*_AH_ ≈ 29.06 *μ*A/cm^2^, so in a narrow range *I*_SN_ < *I* < *I*_AH_ an isolated node is bistable, possessing a stable equilibrium and a stable limit cycle. When the nodes are coupled on a tree network and external currents are applied to the peripheral nodes, the dynamics of the network may become quite complex. For example, in case of weak coupling, peripheral nodes fire APs, which fail to propagate to the central node, so that nodes in the inner generations of the network exhibit small-amplitude spikes. For a stronger coupling, nodes in the inner generations may fire APs, but with skipping relative to APs in the periphery, demonstrating various *m*:*n* synchronization patterns. However, for strong coupling and sufficiently high external currents the network shows fully synchronized periodic firing.

**Figure 2:**
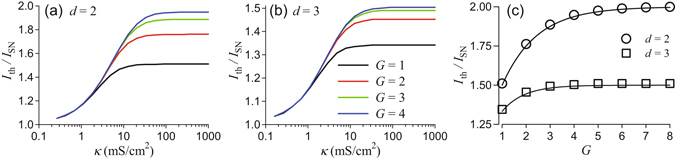
Threshold constant current, *I*_th_, which gives rise to repetitive APs at the central node of a tree network with branching *d* and number of generation, *G*. The threshold current is normalized by the bifurcation value *I*_SN_ = 28.15 *μ*A/cm^2^ at which a single isolated HH node starts to generate a periodic sequence of APs. (**a,b**): Threshold current vs the coupling strength, *κ*, for the indicated number of generations, *G* (see legend on panel b) and the branching, *d* = 2 (**a**) and *d* = 3 (**b**), respectively. (**c**): Threshold constant current resulting from simulations (symbols) vs the number of generations for strong coupling *κ* = 100 mS/cm^2^ and indicated values of branching, *d*. Solid lines show the scaling relation (12).

A comprehensive analysis of the deterministic dynamics is beyond the scope of this study. Instead, since our primary interest is in the emergence of periodic sequences of full-size APs at the central node, we address the following question: Given the tree topology, *G* and *d*, and the coupling strength, *κ*, what is a threshold value *I *_th_ of a constant current applied to peripherals, *I*, which makes the central node to generate repetitive firing of full-size APs? To this end, we perform simulations of tree networks with given *κ*, *G* and *d*. Initially membrane potentials of the individual nodes are randomly distributed around the stable equilibrium of an isolated node for *I* = 0. Then we apply a current *I* > 0 and determine the minimal value, *I *_th_ of *I* at which the central node generates APs repetitively at steady state. Results are shown in Fig. 2(a,b). At a given *κ*, there is periodic firing of APs for values of *I* above the corresponding curves in Fig. 2(a,b). Below these curves, the network is excitable in the sense that no repetitive firing of APs is observed at the central node. In the following, we refer to these two regimes as oscillatory (repetitive firing of full-size APs by the central node) and excitable (no repetitive firing of APs by the central node). The threshold value of the external current, *I*_th_, increases for weak and moderate values of the coupling strength, as coupling acts effectively as a leak current for a node. Consequently the network needs stronger external input to the peripheral nodes to sustain periodic firing of the central node.

Figure 2(a,b) shows two distinct coupling regimes. For weak coupling, *κ* < 2 mS/cm^2^, the threshold current *I *_th_ is independent of the network size, i.e. the number of generations, *G*, and branching, *d*. Since coupling is weak, nodes are only affected by adjacent nodes and only during AP firing. In contrast, for strong coupling, *κ* > 60 mS/cm^2^, the threshold current saturates, and its value increases with increasing number of generations. This is illustrated further in Fig. 2(c) showing the threshold current vs the number of generations for strong coupling. Note that the strong coupling regime spans the range of realistic coupling strengths for models of branched myelinated dendrites. As can be seen in Fig. 2(c), the threshold current follows a characteristic dependence saturating for trees with a large number of generations, *G*, and decreases with the increase of branching, *d*. This results from the fact that networks with larger *d* have more peripheral nodes compared to the total number of nodes. Thus, the input is distributed among more nodes. In order to quantify this effect, we consider the scaling of the threshold current, *I*_th_, with the relative number of peripheral nodes,

11 $$R(G,d)=\frac{{N}_{p}}{N}=\frac{{d}^{G}(d-\mathrm{1)}}{{d}^{G+1}-1},\quad {R}_{\infty }(d):=\mathop{\mathrm{lim}}\limits_{G\to \infty }R(G,d)=\frac{d-1}{d},$$

which follows from Eq. (1). For the threshold current, *I*_th_, we find that the scaling relation,

12 $${I}_{{\rm{th}}}={R}^{-1}(G,d){I}_{{\rm{SN}}},$$

where *I*_SN_ is a bifurcation value of the constant current in the isolated single node, approximates well the threshold current obtained form simulations in Fig. 2(c). We note that the scaling factor, *R*(*G*,*d*), decreases with the number of generation and appoaches its limiting value, *R*_∞_(*G*), already for trees with *G* > 5 generations.

Deterministic trees with the Frankenhaeuser-Huxley nodal model show similar dynamics with the same scaling as in Fig. 2(c). The scaling relation, Eq. (12), holds for strongly-coupled trees and is derived in the following section.

### Stochastic Dynamics

**Figure 3:**
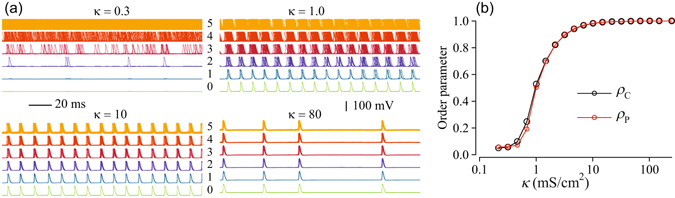
Synchronization of APs firing in a tree with *G* = 5 generations and *d* = 3 branching in the excitable regime. Peripheral nodes were excited by the external currents (4) with parameters *I* = 20 *μA*/cm^2^ and *D* = 500 (*μ*A/cm^2^)^2^ ms. (**a**): Voltage traces of the HH nodes. Each sub-panel shows 200 ms long superimposed voltage traces of nodes within a generation *g*, *g* = 1, …, 5, for the indicated coupling strength, *κ* (mS/cm^2^). Horizontal axis is time. Numbers next to voltage traces indicate generations within the tree, *g* = 0 corresponds to the central node and *g* = 5 corresponds to the peripheral generation, respectively. (**b**): Kuramoto order parameters of the network vs the coupling strength; *ρ*_P_ is calculated for the peripheral nodes only; *ρ*_C_ is calculated for all nodes.

The addition of uncorrelated noise to the peripheral nodes allows for the generation of APs in the excitable regime. Figure 3(a) shows an example of the stochastic dynamics for a tree with *G* = 5 generations and *d* = 3 branching. In the excitable regime (*I* = 20 *μ*A/cm^2^) noise of sufficient intensity induces APs in peripheral nodes. For weak coupling (*κ* = 0.3 mS/cm^2^) noise-induced APs in adjacent generations are not synchronized (superimposed spikes for peripheral nodes fill densely corresponding generation panels) and do not propagate beyond the 2-nd generation, which shows only sparse APs. Increasing the coupling strength leads to progressive synchronization of nodes in adjacent generations, resulting in APs in the central node. The degrees of synchronization of the peripheral nodes and of the entire networks are quantified using the Kuramoto order parameters, *ρ*_P_ and *ρ*_C_, respectively.

As shown in Fig. 3(b), these order parameters increase with coupling strength following close dependencies and reflecting progressive synchronization of APs firing in the network. For *κ* > 10 mS/cm^2^, spiking activity of all nodes is synchronized and both order parameters approach one. We also note that strong coupling leads to slower and more random but synchronous firing of APs. Thus, synchronization leads to a global oscillatory response of the excitable tree, even though the peripheral nodes are excited by independent random inputs.

**Figure 4:**
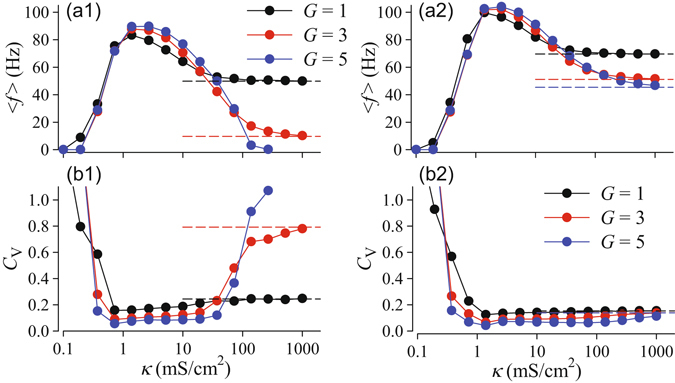
Mean firing rate (a1, a2) and coefficient of variation of interspike intervals (b1, b2) of the central node versus coupling strength for tree networks of HH nodes with *d* = 2 branching for the indicated numbers of generations, *G*. Left panels (a1, b1) correspond to the excitable regime with the constant current *I* = 35 *μ*A/cm^2^; right panels (a2, b2) refer to the oscillatory regime with *I* = 60 *μ*A/cm^2^. The noise intensity is *D* = 500 (*μ*A/cm^2^)^2^ ms. Horizontal dashed lines indicate the theoretical strong coupling limits for an effective node with scaled input current and noise intensity according to Eqs (22) and (23), respectively.

As observed for star networks^42^, the dynamics of the central node in a tree network depends non-monotonously on the coupling strength. As shown in Fig. 4, there exist optimal, rather small values of the coupling strength for excitable and oscillatory trees at which fastest (maximum firing rate) and most coherent (minimal coefficient of variation, *C*_*V*_) firing is observed, respectively. For extremely weak coupling APs, which are fired by different peripheral nodes, are not synchronized and fail to propagate to the central node (Fig. 3, upper left panel). Increasing the coupling strength leads to stronger interaction between the branch nodes and results in synchronous firing of all nodes.

However, the size of a tree, i.e. the number of generations, is critical for the firing statistics of the central node. Furthermore, excitable and oscillatory trees demonstrate qualitatively different behaviour in the biologically-relevant strong coupling regime. In excitable trees, firing of APs becomes slower and more irregular if the coupling is strengthened and trees with more generations are considered. For large *G* and strong coupling firing stops [Fig. 4(a1)] since excitatory inputs to peripheral nodes are too weak to sustain firing of APs. In contrast, in oscillatory trees, the firing rate saturates for strong coupling [Fig. 4(a2)] and firing becomes more regular if strongly-coupled trees with more generations are considered [Fig. 4(b2)]. In order to understand this fundamental difference between excitable and oscillatory trees, we derive a theory for spike generation in strongly-coupled tree networks in the following.

### Scaling of Effective Current and Noise intensity

In the strong coupling regime the dynamics of the central node of a tree network can be described by the dynamics of a single isolated node with membrane potential *V*_0_(*t*) and with effective input current *I*_eff_ and an effective Gaussian noise with intensity *D*_eff_, i.e. the influence of the coupling term on the dynamics can be approximated by a constant current and a white Gaussian noise. Then the dynamics of the membrane potential of the central node in Eq. (2) can be approximated by

13 $$C{\dot{V}}_{0}\approx f[{V}_{0},{{\bf{\text{u}}}}_{0}]+{I}_{{\rm{e}}{\rm{f}}{\rm{f}}}+\sqrt{2{D}_{{\rm{e}}{\rm{f}}{\rm{f}}}}\,{\xi }_{{\rm{e}}{\rm{f}}{\rm{f}}}(t),$$

$${\dot{{\bf{u}}}}_{0}\approx g[{V}_{0},{{\rm{u}}}_{0}\mathrm{].}$$

In the following, we derive those effective parameters for regular trees of diffusively-coupled nodes.

In the network model, the dynamics of the membrane potential of *k*-th node is given by

14 $$C{\dot{V}}_{k}=f[{V}_{k},{{\bf{\text{u}}}}_{k}]+\kappa \sum _{j=0}^{N-1}{A}_{k,j}({V}_{j}-{V}_{k})+{I}_{{{\rm{e}}{\rm{x}}{\rm{t}}}_{{\rm{k}}}},\quad k=0,1,...,N-1.$$

In order to derive approximations for the scaling of the effective current *I*_eff_ and the noise intensity *D*_eff_, we extend the approach of Kouvaris *et al*.^48^, who considered the propagation of excitable waves in a tree network of identical Fitz-Hugh Nagumo nodes in the absence of noisy inputs. Following their approach, we consider the dynamics of the average membrane potential 〈*V*〉_*g*_ (termed density by Kouvaris *et al*.) in each shell in a tree. Here and in the following 〈〉_*g*_ denotes averaging over all nodes of the *g* th shell,

15 $${\langle V\rangle }_{g}:=\frac{1}{{d}^{g}}\sum _{g{\rm{t}}{\rm{h}}\,{\rm{s}}{\rm{h}}{\rm{e}}{\rm{l}}{\rm{l}}}{V}_{k}.$$

The dynamics of those densities can be obtained by averaging the respective equations for the dynamics of the membrane potentials, Eq. (14), over all nodes in one shell. Since the total number of connections between nodes in shell *g* and *g* − 1 is *d* ^*g*^, we obtain

16 $$C{\langle \dot{V}\rangle }_{g}={\langle f(V,{\bf{u}})\rangle }_{g}+\{\begin{array}{ll}\kappa d({\langle V\rangle }_{1}-{V}_{0}), & g=0,\\ \kappa ({\langle V\rangle }_{g-1}-(d+\mathrm{1)}{\langle V\rangle }_{g}+d{\langle V\rangle }_{g+1}), & 0 < g < G,\\ \kappa ({\langle V\rangle }_{G-1}-{\langle V\rangle }_{G})+I+\sqrt{2\frac{D}{{N}_{p}}}\,{\xi }_{G}(t), & g=G\mathrm{.}\end{array}$$

Note that, since peripheral nodes are subject to independent white Gaussian noises, the corresponding equation for the averaged membrane potentials of the peripheral generation contains white Gaussian noise *ξ*_*G*_(*t*) with reduced intensity *D*/*N*_*p*_.

Since the coupling terms depend only on the difference between densities of the membrane potentials in adjacent generations Δ*V*_*g*_: = 〈*V*〉_*g*_ − 〈*V*〉_*g*+1_, we consider the dynamics of those differences next. Subtracting equations for ${\langle \dot{V}\rangle }_{g}$ yields,

17 $$\begin{array}{cc}C{\rm{\Delta }}{\dot{V}}_{g} & =({\langle f(V,{\bf{u}})\rangle }_{g}-{\langle f(V,{\bf{u}})\rangle }_{g+1})+{\rm{\Delta }}{I}_{g}+{\rm{\Delta }}{\xi }_{g}(t)\\  & +\kappa \{\begin{array}{cc}(d{\rm{\Delta }}{V}_{1}-(d+1){\rm{\Delta }}{V}_{0}), & g=0,\\ ({\rm{\Delta }}{V}_{g-1}-(d+1){\rm{\Delta }}{V}_{g}+d{\rm{\Delta }}{V}_{g+1}), & 0 < g < G-1,\\ ({\rm{\Delta }}{V}_{G-2}-(d+1){\rm{\Delta }}{V}_{G-1}), & g=G-1,\end{array}\end{array}$$

where Δ*I*_*g*_=−*δ*_*g*,*G*−1_ *I* and Δ*ξ*_*g*_(*t*) $=-{\delta }_{g,G-1}\sqrt{2\frac{D}{{N}_{p}}}{\xi }_{G}(t)$ is Gaussian white noise.

Next, we consider the case of strong coupling. In that case, Δ*V*_*g*_ becomes small, and the membrane potentials of individual nodes approach the average potentials of the corresponding shell. Thus, we can approximate 〈*f*(*V*, **u**)〉_*g*_ − 〈 *f*(*V*, **u**)〉_*g*+1_ by a Taylor expansion around Δ*V*_*g*_ = 0, i.e. 〈* f*(*V*, **u**)〉_*g*_ − 〈 *f*(*V*, **u**)〉_*g*+1_ ≈ Δ*f*_*g*_^0^ + Δ*f* ′_*g*_Δ*V*_*g*_ + *h*.*o*. It then follows for strong coupling, i.e.

18 $$\Delta {f}_{g}^{0}=\mathrm{0,}\quad \kappa \gg |\Delta {f^{\prime} }_{g}|,g=0,\,1,\,...,\,G-1,$$

that the dynamics of the averaged potential is dominated by the coupling term and Δ*V*_*g*_ can be approximated by a multidimensional Ornstein − Uhlenbeck process,

19 $$C\frac{d}{dt}{\rm{\Delta }}{\bf{V}}\approx \kappa {\bf{B}}{\rm{\Delta }}{\bf{V}}+{\rm{\Delta }}{\bf{I}}+{\rm{\Delta }}\xi (t\mathrm{).}$$

Here we introduced the *G*-dimensional vectors,

$$\begin{array}{cc}{\rm{\Delta }}{\bf{V}} & ={({\rm{\Delta }}{V}_{0},{\rm{\Delta }}{V}_{1},...,{\rm{\Delta }}{V}_{G-1})}^{T},\quad {\rm{\Delta }}{\bf{I}}={({\rm{\Delta }}I,{\rm{\Delta }}{I}_{1},...,{\rm{\Delta }}{I}_{G-1})}^{T},\quad \\ {\rm{\Delta }}{\bf{x}}(t) & \,={({\rm{\Delta }}{\xi }_{0}(t),{\rm{\Delta }}{\xi }_{1}(t),...,{\rm{\Delta }}{\xi }_{G-1}(t))}^{T},\end{array}$$

and the *G* × *G* tridiagonal Toeplitz matrix,

20 $${\bf{B}}=(\begin{array}{ccccc}-(d+1) & d & 0 & ... & 0\\ 1 & -(d+1) & d & ... & ...\\ 0 & 1 & -(d+1) & ... & 0\\ ... & ... & ... & ... & d\\ 0 & .. & 0 & 1 & -(d+1)\end{array}).$$

In the strong coupling limit (18), deviations of Δ**V** from its mean value decay extremely fast and we can use an adiabatic elimination^49^ to approximate Δ**V** by its mean value plus a white Gaussian noise. Both, the mean voltage difference and the intensity of the Gaussian white noise in the strong coupling limit can be obtained by setting the left-hand side of Eq. (19) to zero. This yields

21 $$\Delta {\bf{V}}\approx -\frac{1}{\kappa }{{\bf{B}}}^{-1}({\rm{\Delta }}{\bf{I}}+{\rm{\Delta }}\xi (t)),$$

where **B** ^−1^ is the inverse of the matrix **B**. In order to obtain an approximation for the dynamics of the central node, we can use Eq. (21) to replace *V*_0_ − 〈*V*〉_1_ by Δ*V*_0_ in Eq. (16) for the central node, *g* = 0. This yields

22 $$C{\langle \dot{V}\rangle }_{0}=C{\dot{V}}_{0}=f[{V}_{0},{{\bf{u}}}_{0}]+d{{\bf{B}}}^{-1}{({\rm{\Delta }}{\bf{I}}+{\rm{\Delta }}\xi (t))}_{1}\mathrm{.}$$

Here and in the following the index “1” denotes the first component of a *G*-dimensional vector. Next, the effective parameters *I*_eff_ and *D*_eff_ can be obtained by comparing Eqs (22) and (13). This yields the effective input current and the intensity of the effective white Gaussian noise,

23 $${I}_{{\rm{eff}}}=d{({{\bf{B}}}^{-1}{\rm{\Delta }}{\bf{I}})}_{1},\quad \sqrt{2{D}_{{\rm{eff}}}}\,{\xi }_{{\rm{eff}}}(t)=d{({{\bf{B}}}^{-1}\Delta \xi (t))}_{1}\mathrm{.}$$

For the special case, considered in this study, that only peripheral nodes are subject to noisy inputs, i.e. Δ**I** = (0, 0, …, − *I*)^*T*^, and $\Delta \xi (t)={(0,0,{\rm{\ldots }},-\sqrt{(2D/{N}_{p})}{\xi }_{G}(t))}^{T}$, the calculation of the effective parameters *I*_eff_ and *D*_eff_ requires only a single component, (1,*G*), of the inverse matrix, **B** ^−1^. Since **B** is a tridiagonal Toeplitz matrix, we can apply the results of Ref. 50 to calculate this component (see Supplemental Material for details on calculations) and find for the effective current,

24 $${I}_{{\rm{eff}}}=\frac{{N}_{p}}{N}I=R(G,d)I,$$

and for the effective noise intensity,

25 $${D}_{{\rm{eff}}}=\frac{{N}_{p}}{{N}^{2}}\,D=\frac{R(G,d)}{N}\,D,$$

where the scaling factor *R*(*G*,*d*) is given by Eq. (11). It immediately follows that for large trees (*G* → ∞) the effective current approaches *I*(*d* − 1)/*d* and the effective noise intensity approaches zero.

**Figure 5:**
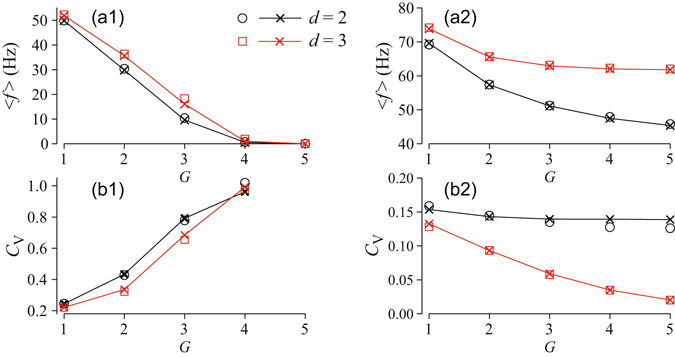
Mean firing rate, 〈* f* 〉, (a1, a2) and coefficient of variation, *C*_*V*_, of interspike intervals (b1, b2) of the central node versus the number of generations of tree networks in the strong coupling regime, *κ* = 1000 mS/cm^2^ and noise intensity *D* = 500 (*μ*A/cm^2^)^2^ ms. Left panels (a1, b1) correspond to an excitable tree with constant current *I* = 35 *μ*A/cm^2^; right panels (a2, b2) refer to an oscillatory tree with *I* = 60 *μ*A/cm^2^. Symbols $\square$ and ○ mark results of numerical simulations of the corresponding network with the indicated branching, *d*; solid lines and symbols × show theoretical scaling predictions.

**Figure 6:**
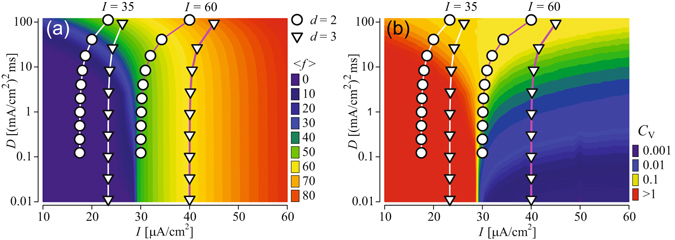
Heat maps of the mean firing rate, 〈* f*〉 (**a**), and coefficient of variation, *C*_*V*_ (**b**), vs input current, *I*, and noise intensity, *D*, for the single isolated HH node obtained from numerical simulations. Symbols and lines show the scaling of the current, Eq. (24), and noise intensity, Eq. (25), respectively, for tree networks with indicated values of the branching *d* (*d* = 2, circles; *d* = 3, triangles) and with increasing number of generations, *G* from 1 (top) to 10 (bottom). These lines correspond to Fig. 5. For tree networks, the input current to the peripheral nodes is *I* = 35 *μ*A/cm^2^ (white lines) and *I* = 60 *μ*A/cm^2^ (magenta lines) and the noise intensity is *D* = 500 (*μ*A/cm^2^)^2^ms.

Investigating the scaling of the effective parameters in more detail, we first note that our theory yields the scaling relation, Eq. (12), observed for the deterministic threshold current in Fig. 2(c). In fact, the same scaling relation applies to the bifurcation values of *I* in the deterministic model, e.g. the subcritical Andronov-Hopf bifurcation of the equilibrium or the saddle-node bifurcation of the limit cycles. Second, in Fig. 5, we demonstrate the validity of the theoretical scaling predictions by comparing results for the mean firing rate and the CV from direct simulation of tree networks with those from a single node (13) with input current and noise intensity scaled according to Eqs (24) and (25), respectively. As illustrated in Fig. 5, we find an excellent correspondence of both results. This indicates that in the strong coupling limit the response of the network can be predicted from the stochastic dynamics of the effective central node. The statistics of interspike intervals for a single isolated node versus input current parameters, i.e. constant component, *I*, and noise intensity, *D*, can be easily computed numerically yielding two-dimensional maps, such as shown in Fig. 6. Then for a given size (number of generations, *G*) and branching, *d*, of a tree, the scaled parameters, Eqs (24) and (25), set an operation point for the tree on the parametric map of a single element. Figure 6 demonstrates this for trees of strongly-coupled HH nodes in the excitable and oscillatory regimes, with input currents to the peripheral nodes *I* = 35 and *I* = 60 *μ*A/cm^2^, respectively. Each point on the map corresponds to a set (*I*_eff_, *D*_eff_) resulting for a tree network. The color code then yields the firing rate and CV of a single node with (*I*,*D*) = (*I*_eff_, *D*_eff_).

In trees with more generations *G*, the operation point is shifted towards smaller currents and lower noise intensities. This results from two aspects. First, as discussed above, trees with more generations have smaller relative numbers of peripheral nodes *R*. Thus, the noisy input current is distributed among more nodes. Therefore, the effective current *I*_eff_ is reduced. For trees with large numbers of generations, *I*_eff_ approaches *I*(*d* − 1)/*d*. Second, the superposition of the independent noises to the peripheral nodes yields an effective noise reduction, so that *D*_eff_ → 0 for trees with large numbers of peripherals *N*_*p*_ = *d* ^*G*^. In combination, both effects cause the qualitatively different behaviour of excitable and oscillatory trees with large numbers of generations in the strong-coupling regime, mentioned in the previous section and illustrated in Fig. 5. In particular, excitable trees approach the well-known weak noise behaviour of excitable systems, i.e. slow and irregular firing. In contrast, AP generation in oscillatory trees occurs at finite firing and becomes more coherent.

Finally, we note that in the strong coupling limit the scaling relations (24, 25) are independent of the particular choice of the nodal model, i.e. they are expected to work for either Hodgkin-Huxley or Frankenhaeuser-Huxley nodal models.

### Signal Detection

The signal detection efficiency of a neuron can be quantified using the discriminability and the Fisher information^47, 51–54^. In case of our model of coupled excitable elements on a tree, we use these measures to characterize how the tree topology affects its ability to distinguish between two stimuli, *I* and *I* + Δ*I*, applied to the peripheral nodes.

The preceding section showed that in the strong coupling limit, the stochastic dynamics of the network could be predicted from the dynamics of a single node with appropriately scaled parameters of the input current. Thus, we first analyze the lower bound of the Fisher information of a single node. Equation (9) indicates that the Fisher information is determined by two factors: the term ${(\frac{d{\mu }_{T}}{dI})}^{2}$, which is related to the slope of the so-called *f* − *I* curve (mean firing rate vs input current curve) and determines the sensitivity of a neuron to small variations of the input current. The sensitivity is largest in the vicinity of the bifurcation point, where the limit cycle is born, and where the slope of the *f* − *I* curve is the steepest. In this region, the Fisher information is high. However, the second factor in Eq. (9), the variance of the spike count, may degrade the Fisher information. In the excitable regime, when the input current is below its bifurcation value and APs are induced by noise, the phenomenon of stochastic resonance is observed^55^, i.e. due to the competition of the two factors, the sensitivity and the spike count variance, the Fisher information possesses a maximum at an optimal noise intensity^47^.

**Figure 7:**
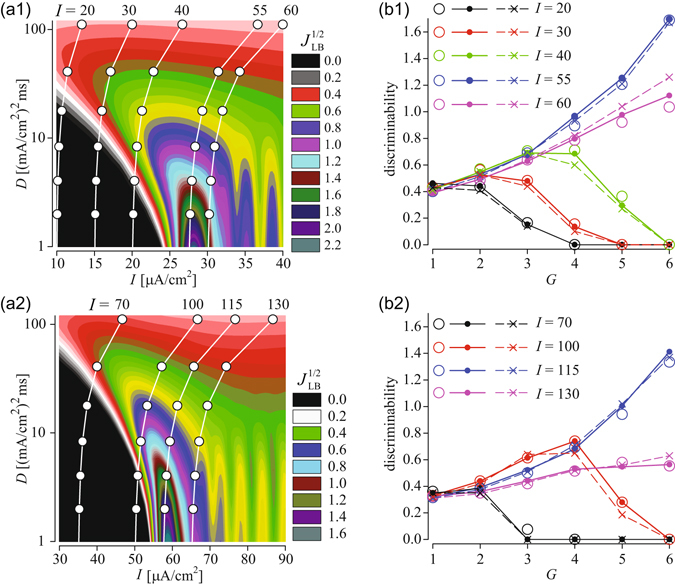
Signal detection by a single node and by tree networks. (**a**): Square root of the lower bound of the Fisher information (9) of the single HH (a1) and single FH (a2) node versus input current and noise intensity. Superimposed white lines with circle symbols show the scaling of the effective input current, Eq. (24), and effective noise intensity, Eq. (25), for tree networks with branching ratio *d* = 2 and increasing number of generations. Each of these lines corresponds to values of the external current to peripheral nodes, *I*, indicated at the top of panels (**a**). The number of generations, *G*, increases from 1 (top) to 6 (bottom). Noise intensity for tree networks is *D* = 500 [(*μ*A/cm^2^)^2^ms]. (**b**): Discriminability (8) versus the number of generations, *G*, for tree networks with branching ratio *d* = 2 and indicated values of the input current *I* for HH (b1) and FH (b2) nodes. The increment of the input current is Δ*I* = 2 *μ*A/cm^2^ and noise intensity is *D* = 500 [(*μ*A/cm^2^)^2^ms]. Open circles refer to results from numerical simulations of corresponding tree networks. Solid lines with filled circles show the theoretical scaling predictions obtained from numerical calculations of the discriminability of a single HH or FH node with input current and noise intensity scaled according to Eqs (24) and (25), respectively. Dashed lines with symbols × show the theoretical predictions of discriminability obtained from the lower bound of the Fisher information of panels (a1, a2) using relation (10) with the input current increment scaled according to Eq. (24). In all panels the spike count statistics was calculated for *T* = 200 ms windows. The coupling strength on the panels (b1, b2) is *κ* = 1000 mS/cm^2^.

Figure 7(a1) shows the lower bound of the Fisher information, *J*_LB_, for a single HH node as a function of input current and noise intensity. The Fisher information is maximal for an input current *I*, which brings the system close to the transition to periodic spiking, i.e. 28–29 *μ*A/cm^2^. In Fig. 7(a1), a vertical section across the map corresponds to the dependence of the Fisher information on noise intensity. As can be seen, such a dependence is non-monotonous in the excitable regimes, e.g. for *I* = 15 or *I* = 20 *μ*A/cm^2^, indicating the phenomenon of stochastic resonance^55^, reported before for the original Hodgkin-Huxley neuron model in Ref. 47 Indeed, stochastic resonance is a generic phenomenon in excitable systems^20, 55^ and so the Frankenhaeuser-Huxley (FH) nodal model demonstrates qualitatively similar parameter dependence, shown in Fig. 7(a2). In the absence of noise, a stable equilibrium of the single FH node passes through a subcritical Andronov-Hopf bifurcation at *I*_AH_ ≈ 60.19 *μ*A/cm^2^. Consequently, the Fisher information in Fig. 7(a2) is maximal around this value, similar to the HH node. In the excitable regime, e.g. *I *= 40 *μ*A/cm^2^, the Fisher information vs. noise intensity passes through a maximum, demonstrating stochastic resonance, again, qualitatively similar to the HH node.

The scaling relations for the input current and noise, Eqs (24,25), in combination with the relation between the discriminability and the lower bound of the Fisher information (10), enable us to predict the signal detection capability of tree networks in the regime of strong coupling. Given the branching, *d* and the input current to the peripheral nodes, an increase in the number of generations (i.e. tree size) results in a decrease of the effective input current and noise. Then, depending on the particular values of *I* and *D*, signal detection by the tree may show distinct dependencies on the tree size, *G*. This is illustrated in Fig. 7(a1,a2) by superimposing the scaling of the input current and noise intensity on the Fisher information map of the single node. In particular, our theory predicts that in the excitable regime, i.e. when the network does not produce sustained periodic firing in the absence of stochastic inputs, the scaling of *I* and *D*, may bring an effective operating point of the network across the local maximum of the Fisher information, which should result in larger values of the discriminability for corresponding tree network. As can be seen, for instance, for the input current *I* = 30 or 40 *μ*A/cm^2^ for HH nodal model, an increase of the number of generations to *G* = 2–4 brings the effective operating point to regions of higher values of the Fisher information; further growth of the tree size eventually suppresses AP firing and thus small signals cannot be detected. In contrast, in the oscillatory regime (e.g. for *I* = 60 *μ*A/cm^2^ for HH nodal model), the increase of the network size moves the operation point always to regions of higher values of the Fisher information and so the discriminability increases monotonously with the tree size, *G*. Interestingly, one could predict the input current to the network which for a tree with large enough generations would result in an effective operating point close to bifurcation value of the single node. For example, for the HH nodes, such a value of the external current is *I* = 55 *μ*A/cm^2^ and for the FH node, *I* = 115 *μ*A/cm^2^. For such currents increasing the tree size should result in a higher degree of signal discrimination.

To test these predictions we compare the discriminability, Eq. (8), of tree networks to that of the single node representation Eq. (13) with appropriately scaled current and noise intensity, Eqs (24) and (25), respectively. We also calculated the discriminability estimated from the lower bound of the Fisher information (10) from the maps in Fig. 7(a1,a2), with the scaled stimulus increment, $d^{\prime} =R(G,d){\rm{\Delta }}I{\sqrt{{J}_{LB}}}^{1/2}$. Figure 7(b1,b2) shows good correspondence between the theoretical and numerical discriminabilities.

## Conclusion

We have studied the emergence of noisy periodic spiking in regular tree networks of coupled excitable elements. Using biophysical models of excitable nodes, we showed that noisy periodic network spiking can be generated, although the periphery of the tree is excited by random and independent inputs (Fig. 3). The firing rate and coherence of spiking can be maximized by varying the coupling strength and is altered by changing the network topology (Figs 4,5).

We put special emphasis on the strong coupling regime, which refers to the case of excitable nodes of Ranvier linked by myelinated (dendrite or axon) fibers of a neuron. It is intuitively clear that in the strong coupling limit, the collective dynamics of the network could be described by a single effective excitable system. We have derived the corresponding scaling relations for random inputs Eqs (24, 25) which allows for reliable predictions of the collective network response based on the stochastic dynamics of a single isolated node with scaled input parameters. Stochastic excitable systems demonstrate non-trivial behaviour versus the noise intensity. Examples include the phenomena of coherence resonance^56^, whereby the variability of spiking events (e.g. coefficient of variation) is minimal for non-zero noise intensity, and stochastic resonance, characterized by non-monotonous dependence of a response to an external signal on the noise intensity^55^. Similar phenomena have been observed in networks of excitable elements. In particular, the phenomena of system size stochastic^57^ and coherence resonance^22^, which are also observed in strongly-coupled star networks of excitable elements^42^. As we have shown in the present paper, the phenomenon of system size stochastic resonance also occurs in strongly coupled tree networks, i.e. the number of generations in a tree network of excitable elements can be tuned in order to optimize the network ability to discriminate between different input signals. In particular, our analytical approach allows for the prediction of optimal tree sizes and branching ratios.

The analytical approach developed here for the strong coupling can be extended to random trees^58^ in which the branching ratio varies among different generations, yielding similar scaling relations in the strong coupling limit. While we considered networks of identical nodes, our approach can be readily extended to the inhomogeneous case, as long as the condition for strong coupling Eq. (18) is satisfied. Inhomogeneity in applied currents and/or noise intensity is also easily treatable using Eq. (23). Here, only the average current and noise intensity in each generation contributes, which indicates that strongly coupled tree networks effectively average over inputs, applied at the same distances to the central node.

Our results suggest a mechanism for the emergence of noisy periodic firing and information coding by peripheral sensory neurons which possess branched tree-like myelinated dendrites^36^. Such neurons may possess multiple spike initiation zones at peripheral nodes (heminodes) and nodes of Ranvier at branching points. Examples of the muscle spindles^35^ and cutaneous mechanoreceptors^39^ indicate that myelinated dendritic trees extend to up to 7 generations. Myelin provides low-resistance links between nodes and fast saltatory conduction of APs, allowing for reliable coding and nonlinear integration of external stimuli from spatially extended receptive areas^36^. Myelinated segments correspond to strong coupling between the nodes of Ranvier. For example, diameters of a cat muscle spindles afferents ranges from 3 to 13 *μ*m, while links between nodes are relatively short, 50–200 *μ*m^35^. An estimate of the coupling strength from Eq. (3) yields values well within the range of the strong coupling regime used in our study. The collective noisy periodic firing then may occur due to the synchronized noise-induced generation of APs by stimulating the peripheral heminodes, as described by our model. Given the biophysical properties of the nodes of Ranvier and the sensory inputs, the variability of interspike intervals and the stimulus discrimination capability of a neuron are mainly determined by the ratio of the number of signal-receiving peripheral heminodes to the total number of nodes in the network.

## Electronic supplementary material

Supplementary Information
