# Supplementary material for: Emergent stochastic oscillations and signal detection in tree networks of excitable elements
Source: Sci Rep. 2017 Jun 21;7:3956. doi: 10.1038/s41598-017-04193-8 (PMC5479816; doi:10.1038/s41598-017-04193-8)
Supplement: Supplementary file 1 — Supplementary Information [file 41598_2017_4193_MOESM1_ESM.pdf]

Supplementary material.

Emergent stochastic oscillations and signal detection in regular tree networks of strongly coupled excitable elements

Justus A. Kromer, Ali Khaledi-Nasab, Lutz Schimansky-Geier, Alexander B. Neiman

Models for Nodes of Ranvier

A Hodgkin-Huxley type model (HH) for a node of Ranvier contains only sodium and leak ionic currents. Thus, in eq.(2) of the main paper, the ionic current becomes $I_{\text{ion}} = I_{\text{Na}} + I_{\text{L}}$. For the sodium current we used the Hodgkin-Huxley (HH) type kinetics[1,2], $I_{\text{Na}} = g_{\text{Na}} m^3 h (V - V_{\text{Na}})$, where $g_{\text{Na}} = 1100$ mS/cm^2 is the maximal value of the sodium conductance and $V_{\text{Na}} = 50$ mV is the Na reversal potential. The gating activation and inactivation variables obey the dynamics

$$\begin{aligned} \dot{m} &= \alpha_m(V)(1-m) - \beta_m(V)m \\ \dot{h}_k &= \alpha_h(V)(1-h) - \beta_h(V)h, \end{aligned} \tag{S1}$$

with the following rate functions:

$$\begin{aligned} \alpha_m(V) &= 1.314(V+20.4)/[1-\exp[-(V+20.4)/10.3]], \\ \beta_m(V) &= -0.0608(V+25.7)/[1-\exp[(V+25.7)/11]], \\ \alpha_h(V) &= -0.068(V+114)/[1-\exp[(V+114)/11]], \\ \beta_h(V) &= 2.52/[1+\exp[-(V+31.8)/13.4]]. \end{aligned} \tag{S2}$$

The leak current is $I_{\text{L}} = g_{\text{L}}(V_k - V_{\text{L}})$ with $g_{\text{L}} = 20$ mS/cm^2 and $V_{\text{L}} = -80$ mV.

The Frankenhaeuser-Huxley (FH) model[3] uses four ionic currents: sodium, potassium, persistent sodium and leak: $I_{\text{ion}} = I_{\text{Na}} + I_{\text{K}} + I_{\text{p}} + I_{\text{L}}$. The currents are given by

$$\begin{aligned} I_X &= P_X \frac{EF^2}{RT} \frac{[X]_0 - [X]_i e^{\frac{EF}{RT}}}{1 - e^{\frac{EF}{RT}}}, \quad X = \text{Na, K, p}, \\ I_{\text{L}} &= g_{\text{L}}(V - V_{\text{L}}), \end{aligned} \tag{S3}$$

where $E = -70 + V$; P_X are the permeabilities of sodium (Na), potassium (K) and persistent (p) ionic currents:

$$P_{\text{Na}} = \bar{P}_{\text{Na}} h m^2, \quad P_{\text{K}} = \bar{P}_{\text{K}} n^2, \quad P_{\text{p}} = \bar{P}_{\text{p}} p^2, \tag{S4}$$

where $\bar{P}_X$ are maximal values of permeabilities of corresponding channels. In Eq. (S4) $[X]_0$ and $[X]_i$ are corresponding extracellular and intracellular ionic concentrations, of Na and K ions, respectively; for the persistent current, $X = p$, we have $[X] \equiv [\text{Na}]$. The gating variables follow,

$$\begin{aligned} \dot{m} &= [\alpha_m(V)(1-m) - \beta_m(V)m]s, \\ \dot{h} &= [\alpha_h(V)(1-h) - \beta_h(V)h]s, \\ \dot{n} &= [\alpha_n(V)(1-n) - \beta_n(V)n]s, \\ \dot{p} &= [\alpha_p(V)(1-p) - \beta_p(V)p]s, \end{aligned} \tag{S5}$$

and the rate functions are:

$$\begin{aligned} \alpha_m &= 0.36(V-22)/(1-\exp[(22-V)/3]), \\ \beta_m &= 0.4(13-V)/(1-\exp[(V-13)/20]), \\ \alpha_h &= 0.1(10+V)/(\exp[(V+10)/6]-1), \\ \beta_h &= 4.5/(1+\exp[(45-V)/10]), \\ \alpha_p &= 0.006(V-40)/(1-\exp[(40-V)/10]), \\ \beta_p &= 0.09(25+V)/(\exp[(V+25)/20]-1), \\ \alpha_n &= 0.02(V-35)/(1-\exp[(35-V)/10]), \\ \beta_n &= 0.05(10-V)/(1-\exp[(V-10)/10]), \end{aligned}$$

where s is a scaling factor. The parameters for the model were taken from the original Frankenhaeuser-Huxley paper[3] with three modifications to reduce the frequency of periodic spiking when a sufficiently-high constant current is injected: (i) the rate equations for the gating variables included a scale factor $s = 0.3$; (ii) the maximal permeability of potassium channels is reduced to $\bar{P}_{\text{K}} = 3.6 \times 10^{-4}$ cm/sec; and (iii) the leak conductance was reduced to $g_{\text{L}} = 6$ mS/cm^2. Other parameters are the same as in the original FH model: $[Na]_0 = 114.5$ mM, $[Na]_i = 13.74$ mM, $[K]_0 = 2.5$ mM, $[K]_i = 120$ mM; $\bar{P}_{\text{Na}} = 8 \times 10^{-3}$ cm/sec; $\bar{P}_{\text{p}} = 54 \times 10^{-5}$ cm/sec; $V_{\text{L}} = 0.026$ mV. Constants R and F are the universal gas constant and the Faraday constant, and $T = 293.15$ K.

Inverse of Tridiagonal Toeplitz Matrix

In order to calculate the effective current I_{mod} eq. (20) and noise intensity D_{mod} eq. (21), we need to evaluate the inverse matrix $\mathbf{B}^{-1}$ of $\mathbf{B}$. To this end, we apply results from Ref.[4] in which the components of the inverse of a tridiagonal Toeplitz matrix are given in terms of two sequences $\{v_i\}$ and $\{u_i\}$ with $i = 1, 2, ..., G$. If we apply their results to the matrix $\mathbf{B}$, eq. (17), we obtain for its (i, j)th component

$$\mathbf{B}_{ij}^{-1} = u_{\text{Min(i,j)}} v_{\text{Max(i,j)}} \begin{cases} \frac{d^i}{d^j} & \text{if } i < j \\ 1 & \text{else} \end{cases} . \tag{S6}$$

The sequences $\{u_i\}$ and $\{v_i\}$ are only defined up to a multiplicative constant and it is convenient to set $u_1 = 1$. Then the sequences can be calculated from

$$v_1 = \frac{1}{d_1},\ v_k = -\frac{d}{d_k} v_{k-1}, \quad k = 2, 3, .., G$$

with

$$d_G = -(d+1),\ d_i = -(d+1) - \frac{d}{d_{i+1}}, \quad i = 1, ..., G-2, G-1$$

and

$$u_G = \frac{1}{w_G v_G},\ u_k = -\frac{d}{w_k} u_{k+1}, \quad k = 1, ..., G-2, G-1$$

with

$$w_1 = -(n_1 + 1),\ w_i = -(d+1) - \frac{d}{w_{i-1}}, \quad i = 2, 3, .., G.$$

Since we are interested in the dynamics of the first order node ($g = 0$) when only peripherals are subject to noisy currents, we only need to evaluate a single component, $(1, G)$, of $\mathbf{B}^{-1}$. For this component we find from eq. (S6), $\mathbf{B}_{1,G}^{-1} = v_G$. Applying the definition of v_k, eq. (S7), multiple times, we obtain

$$v_G = (-1)^{G-1} \frac{d^{G-1}}{\prod_{k=1}^{G} d_k}. \tag{S7}$$

Next, we evaluate the product for which one can show by using mathematical induction that

$$\prod_{k=1}^{j} d_k = (-1)^j \left(\sum_{k=0}^{j} d^k - \frac{d}{d_{j+1}} \sum_{l=0}^{j-1} d^k \right), \quad j < G. \tag{S8}$$

Finally, multiplication by d_G and applying eq. (S7) yields

$$\prod_{k=1}^{G} d_k = (-1)^G \sum_{l=0}^{G} d^l = (-1)^G N. \tag{S9}$$

This yields for the $(1, G)$ component of $\mathbf{B}^{-1}$

$$\mathbf{B}_{1G}^{-1} = -\frac{d^{G-1}}{N}. \tag{S10}$$

Using this in eqs. (19) yields the effective current and noise intensity, respectively.

References

1. McIntyre, C. C., Richardson, A. G. & Grill, W. M. Modeling the excitability of mammalian nerve fibers: influence of afterpotentials on the recovery cycle. *Journal of Neurophysiology* **87**, 995–1006 (2002).
2. Hodgkin, A. L. & Huxley, A. F. A quantitative description of membrane current and its application to conduction and excitation in nerve. *The Journal of Physiology* **117**, 500 (1952).
3. Frankenhaeuser, B. & Huxley, A. The action potential in the myelinated nerve fibre of xenopus laevis as computed on the basis of voltage clamp data. *The Journal of Physiology* **171**, 302 (1964).
4. Da Fonseca, C. & Petronilho, J. Explicit inverses of some tridiagonal matrices. *Linear Algebra and its Applications* **325**, 7–21 (2001).
